# FAIR-Checker: supporting digital resource findability and reuse with Knowledge Graphs and Semantic Web standards

**DOI:** 10.1186/s13326-023-00289-5

**Published:** 2023-07-01

**Authors:** Alban Gaignard, Thomas Rosnet, Frédéric De Lamotte, Vincent Lefort, Marie-Dominique Devignes

**Affiliations:** 1 Nantes Université, CNRS, INSERM, l’institut du thorax, F-44000 Nantes, France; 2 TAGC/INSERM U1090, Univ Aix-Marseille, Marseille, France; 3 UMR AGAP Institut, Univ Montpellier, CIRAD, INRAE, Institut Agro, F-34398 Montpellier, France; 4 LIRMM, Univ Montpellier, CNRS, Montpellier, France; 5 Université de Lorraine, CNRS, Inria, LORIA, F-54500 Nancy, France; 6 Institut Français de Bioinformatique, CNRS UAR 3601, Évry, France

**Keywords:** FAIR, Schema.org, Bioschemas, SPARQL, SHACL

## Abstract

The current rise of Open Science and Reproducibility in the Life Sciences requires the creation of rich, machine-actionable metadata in order to better share and reuse biological digital resources such as datasets, bioinformatics tools, training materials, *etc.* For this purpose, FAIR principles have been defined for both data and metadata and adopted by large communities, leading to the definition of specific metrics. However, automatic FAIRness assessment is still difficult because computational evaluations frequently require technical expertise and can be time-consuming. As a first step to address these issues, we propose *FAIR-Checker*, a web-based tool to assess the FAIRness of metadata presented by digital resources. *FAIR-Checker* offers two main facets: a “Check” module providing a thorough metadata evaluation and recommendations, and an “Inspect” module which assists users in improving metadata quality and therefore the FAIRness of their resource. *FAIR-Checker* leverages Semantic Web standards and technologies such as SPARQL queries and SHACL constraints to automatically assess FAIR metrics. Users are notified of missing, necessary, or recommended metadata for various resource categories. We evaluate *FAIR-Checker* in the context of improving the FAIRification of individual resources, through better metadata, as well as analyzing the FAIRness of more than 25 thousand bioinformatics software descriptions.

## Introduction

The production of scientific data intensifies from year to year, leading to a huge, ever-growing amount of data. Open Science is currently seen by many scientific communities and research funding organisations as a way to considerably simplify meta-analyses and improve the statistical power and validity of scientific models. It also contributes to more cumulative and reproducible science by speeding up the development of new hypotheses, data analysis methods, models, and their validation on various open and available datasets, as illustrated in the neuro-imaging community [1] or more recently during the Covid-19 health crisis [2, 3]. Open Science also appears to be an essential factor in limiting the energy and environmental impact of data production and storage, by limiting unnecessary duplication of data and experiences [4].

In practice, Open Science initiatives require digital resources (data, tools, registries, ontologies, etc.) to be findable (F), accessible (A), interoperable (I) and reusable (R). The FAIR fundamental guiding principles have been defined in 2014 by the FORCE-11 group and published in 2016 [5]. However, there was at that time no unified recommendation about how to implement these foundational principles. Rapidly after, the need has emerged to evaluate and assess the FAIR maturity of digital resources [6]. Several communities have produced alternative documents to guide implementation choices and converged during the last few years  [7]. In this paper, we particularly refer to the FAIR Data Maturity Model Specification and Guidelines published in 2020 by the Research Data Alliance^1^ and to the convergent interpretation by the GO FAIR foundation^2^.

Initially, the FAIR evaluation of a resource or a set of resources was mainly done through questionnaires (which have the disadvantage of being time-consuming and requiring a certain amount of experience) . Rapidly enough, automatic tools have been developed, relying on various implementations of the FAIR principles. The first one was the FAIR Evaluation Services^3^ [6], but it was rapidly followed by FAIR-shake^4^[8] and F-UJI^5^ [9].

The scope of the FAIR principles is very broad and therefore impacts a wide range of scientific communities. For example, an expert in microscopy image analysis needs to share both analysis algorithms and reference datasets with perennial identifiers and a controlled vocabulary specific to his or her community. Biologists feeding a genomics database must be able to describe the provenance of their sequences and samples. These same questions arise in the context of the study of marine biodiversity, for example. Thus, a facilitated access to a wide variety of semantic tools is needed if we want to engage a wide variety of communities towards FAIRer resources.

Semantic web technologies seem to respond precisely to this need. Although some *Findability* and *Accessibility* principles are difficult to implement with Semantic Web technologies, such as long-term persistence of identifiers, or indexing by Web search engines (if we leave aside Google Dataset Search), most of the *Interoperability*, and *Reuse* principles can actually be addressed with already established Semantic Web standards technologies.

In this paper, we propose *FAIR-Checker* as a tool, leveraging Knowledge Graphs and Semantic Web technologies, with the goal of making producers and developers of scientific digital resources more accountable and efficient in their FAIR implementation. The main contributions of this paper are *i)* a collection of SPARQL queries aimed at evaluating FAIR principles for metadata, *ii)* a SHACL constraints generator aimed at evaluating metadata profiles and thus enhancing the completeness of metadata, and *iii)* an evaluation of our approach against typical bioinformatics resources.

The paper is organized as follows. Section “Background” describes use cases and the state of the art. Our approach is described in section “Approach”. The system implementation and its evaluation are reported in sections “Implementation” and “Results”. Section “Discussion and conclusion” finally discusses our results and their perspectives.

## Background

### Motivating scenarios

Our main motivation for developing a new FAIR maturity assessment tool was to meet user needs and go beyond a simple scoring system. Improving the discoverability, interoperability, and reuse of a biological resource requires specific technical skills and an understanding of FAIR principles. We wanted to develop a tool based on Knowledge Graphs that facilitates the selection of appropriate ontology terms to enrich the metadata of web resources. Working in the context of a research infrastructure for bioinformatics developing services for health, plant sciences and agronomy communities, we identified 3 typical user profiles: *data producers*, *software developers*, and *repository developers*. These users typically use the web as an infrastructure to access and publish FAIR digital scientific resources. Most of the time, these people are not trained to implement the FAIR principles and have to learn how to do it on the job. As a motivation, we consider the following three use cases.

*UC*$_1$*: Selecting a data repository.* In this use-case, data producers want to select a public data repository (e.g. Dataverse or Zenodo for instance) for publishing a given dataset. They can use *FAIR-Checker* to compare the FAIR compliance of several solutions. To do so, they simply need to submit to *FAIR-Checker* randomly selected identifiers of any dataset hosted by the repositories and compare *FAIR-Checker* analysis reports. This is very fast and does not require any particular semantic web expertise.

*UC*$_2$*: Sharing a software.* Software developers wishing to provide access to a particular tool on the web can create an early version of their website and submit its provisional URL for analysis by *FAIR-Checker*. The analysis report will provide not only an initial FAIRness assessment of their resource, but also recommendations to improve its compliance with the FAIR principles. After making the necessary changes, the developers will be able to test their website again and see the improvements. In such a use-case, specific help is expected regarding the selection of appropriate classes and properties to enrich the metadata. Indeed, relevant ontologies can be very complex to comprehend for developers who are not familiar with.

*UC*$_3$*: Enriching metadata for registries.* Repository developers maintaining a catalog of web-accessible resources may wish to enrich the metadata content associated with each web page in their catalog. They may take advantage of metadata profiles adapted to their community needs. They will submit several pages to *FAIR-Checker* to inspect the quality of associated metadata. *FAIR-Checker* should return useful advice on how to comply with existing metadata profiles. The repository developers can then easily propagate the suggested enriched metadata through the whole catalogue.

### Related works

A rapid survey of existing tools is necessary to assess whether any of them can provide a solution for the three use-cases.

A first group of tools consist of questionnaires for manual assessment of FAIR metrics. One can first cite FAIR AWARE from FAIRsFAIR^6^ which is a simple on-line questionnaire which helps researchers and data managers assess how much they know about FAIR requirements for datasets. Thus it cannot be used to score and compare resources with respect to their compliance with FAIR principles as in UC$_1$. Other questionnaires have been compared in 2019 by the RDA FAIR Data Assessment Working Group [10] showing a great heterogeneity in the way questions are expressed for the same principles (note that this preliminary work certainly inspired the RDA guideline document [11] released in 2020).

In the RDA 2019 survey, 12 tools were studied including 11 questionnaires and only one automated tool (FAIR Evaluator see below). From these 11 questionnaires only 3 have evolved to free on-line resources still available today. The Australian ANDS-NECTAR-RDS-FAIR data assessment tool has become the ARDC FAIR data self assessment tool proposed by the Australian Research Data Commons^7^. It provides a FAIR indicator represented by a more-or-less fully colored green bar without any quantification. Moreover, no help is provided for improving the compliance to FAIR principles as expected in UC$_2$. The two Dutch tools: DANS-Fairdat and DANS-Fair enough are now merged in a single tool SATIFYD^8^ (Self-Assessment Tool to Improve the FAIRness of Your Dataset) which contains 12 questions distributed on the four FAIR categories. The answers are visualized by coloring more or less the corresponding letter and providing a percentage of compliance for each category. Moreover, suggestions for improving the FAIRness are provided when the score is not 100%. In addition to these few online questionnaires, the RDA-SHARC community (SHAring Rewards and Credit) also proposes a questionnaire [12] in the form of a spreadsheet downloadable from Zenodo 9, to be completed by researchers. A quantitative score is computed based on the levels of importance (*essential, recommended, desirable*) for each criterion and on four possible answers (*Never/NA, If mandatory, Sometimes, Always*).

In summary, manual FAIR assessment through questionnaires has a great value for training and propagating good understanding of FAIR principles. It should be noted that the field has evolved a lot and many early questionnaires are no more available on-line as they required constant updating of FAIR principles and FAIR metrics definition. Regarding our use-cases however, questionnaires are clearly not the right solution mostly because manual answering would be tedious for UC$_1$, but also because it would require a good prior knowledge of the FAIR principles by the software developer in UC$_2$, and because in UC$_3$, the resource publisher needs a detailed analysis of the metadata content associated with the web pages of his catalog which cannot be provided by a questionnaire.

The second group of FAIR assessment tools are automatic tools which implement FAIR Maturity Index metrics. The FAIR Evaluator system is the result of a community-driven effort, promoted by the GO-FAIR initiative^10^ with the goal of defining FAIR Metrics, soon renamed FAIR Maturity Indicators (MI), and to develop an automatable framework to discover, access and interpret the content of a data resource [6]. Today, the online FAIR-Evaluator front-end^11^ offers the possibility to import MI tests, to create collections of tests and to evaluate a given resource. An API is also available to access the tests through custom programs. Regarding the online tool, each data resource has to be tested individually, user needs to provide an identifier such as ORCID for example. Results are provided with full log information, which is particularly useful when a FAIR MI test has failed, in order to understand what is wrong in the tested resource. However, no recommendation is given to fix the problem and improve the resource. FAIR-shake [8] is a web tool providing both manual and automated assessment. Users start by creating projects to group several resources to be tested. Digital objects are then created, described through a form, and associated to one or more manual or automated metrics, provided by user submitted scripts. A gradient colored matrix (FAIR insignia) is then displayed as a summary of the assessments results. F-UJI [9] is a fully automated testing tool. It focuses on domain-agnostic metadata which can either be embedded into the resources under assessment or provided by external services. F-UJI allows to test ressource URLs or DOIs and generates a graphical summary of the score as well as a detailed technical report of the evaluations. Other tools such as FOOPS! [13] or O’FAIRe [14] have recently been proposed with a focus on the evaluation of semantic digital resources, with specific metrics tailored to the evaluation of computational ontologies.

In summary, automated tools are key to empower non-specialists in providing FAIRer digital scientific resources. They are also key to systematically evaluating and comparing the FAIRness of these resources at web scale. However, the reviewed approaches only partly answer the user needs identified in use-cases $UC_{1-2-3}$. First, they do not yet provide technical recommendations to guide resource publisher in implementing failed assessments. Then, they do not propose to inspect the quality of metadata with respect to the use of community agreed ontologies ($UC_2$), as well as the level of metadata completeness with respect to community profiles ($UC_3$).

**Table 1:** Short description of the major FAIR principles used by *FAIR-Checker*. Adapted from GO-FAIR initiative (https://www.go-fair.org/fair-principles/, consulted in 2022) and RDA guidelines (doi:10.15497/rda00050, 2020)

Abbreviation	Short description (from GO-FAIR)	Priority (from RDA)
F1A	(Meta)data are assigned a globally unique identifier	Essential
F1B	(Meta)data are assigned a persistent identifier	Essential
F2A	Data are described with structured metadata	Essential
F2B	Data are described with metadata populated using shared vocabularies	Essential
A1.1	(Meta)data are retrievable by their identifier using a standardised communication protocol: open, free, and universally implementable	Important(for data)/Essential for metadata
I1	(Meta)data use a formal, accessible, shared, and broadly applicable language for knowledge representation.	Important
I2	(Meta)data use vocabularies that follow FAIR principles	Important
I3	(Meta)data include qualified references to other (meta)data	Useful
R1.1	(Meta)data are released with a clear and accessible data usage license	Essential
R1.2	(Meta)data are associated with detailed provenance	Important
R1.3	(Meta)data meet domain-relevant community standards	Essential

## Approach

### FAIR-checker metadata analysis workflow

**Fig. 1:**
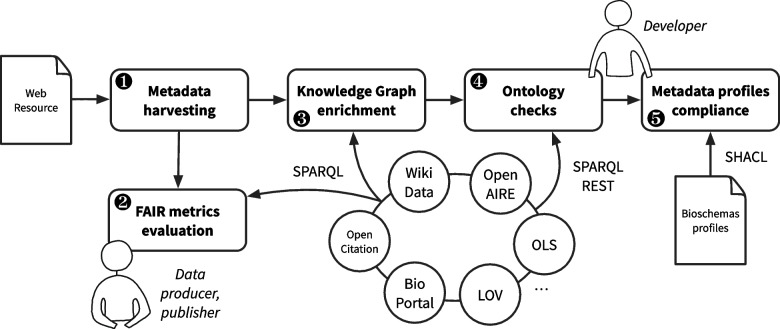
Gathering, enriching and analyzing semantic web annotations in line with FAIR principles

By embedding RDF triples into web pages through JSON-LD, RDFa or HTML microdata, web data providers can semantically advertise search engines with metadata describing the content of web resources. This is particularly attractive to technically comply with the FAIR principles. The general idea of *FAIR-Checker* is to promote the use of embedded metadata in web pages to ease the findability and reuse of digital scientific resources. Figure 1 drafts the main steps for gathering, enriching, and analyzing Semantic Web annotations while benefiting from public Knowledge Graphs.

Given a web page URL, the very first step consists in extracting semantic annotations, based on JSON-LD, RDFa, or HTML microdata standards (➊). This constitutes a minimal starting Knowledge Graph (KG) which is queried with SPARQL for FAIR assessment (➋). Then, for each metadata entity (e.g. a person, a dataset, a software, etc.), public KGs are queried to retrieve relevant associated RDF triples (➌). Since attribution or citation is a clear incentive to promote sharing and reuse in open sciences, we principally target scientific literature KGs such as OpenAire or OpenCitation. We also include general knowledge through WikiData. Following the Linked Data principles, our objective is, i) for data publisher, to limit their efforts in annotating individual web pages, and ii) for Knowledge Graph developers/maintainers to make them contribute to the implementation of FAIR principles. Then, ontology checks (➍) assess that used ontology classes or properties are part of community agreed standards. By leveraging community-specific registries such as BioPortal^12^ or OLS^13^, or general registries such as LOV^14^, we can evaluate if common ontology terms are reused. Finally, since more and more web resources are annotated with Schema.org [15], we leverage the Bioschemas [16] community-agreed profiles to assess the completeness of semantic annotations (➎). These Bioschemas profiles are automatically transformed into SHACL constraints. These constraints are used to indicate the missing triples, considered as mandatory or recommended by the community to describe a certain type of resource. This finally provides users with guidelines for improving the quality of metadata.

### Evaluating FAIR metrics with SPARQL query templates

In this section, we show how SPARQL queries can instrument and operationalize numerous FAIR principles for metadata.

Many of these principles rely on the availability of web-accessible, machine-readable metadata, grounded on community-agreed and shared vocabularies. Being able to automatically parse embedded RDF triples already ensures that metadata is accessible through an open protocol (*Accessibility* principle A1.1) and a structured data format, allowing knowledge representation (*Findability* principle F2, *Interoperability* principle I1). Please refer to Table 1 for FAIR principle brief description.

In our workflow (Fig. 1), SPARQL queries are also used in step 4 to check whether RDF entities match ontology properties present in available registries. This helps in assessing FAIR principles I2 and R1.3. We identified in Table 2 a list of common ontology properties that should be used when publishing FAIR resources.

**Table 2:** Summary of the selected ontology properties relevant to assess three specific FAIR principles in *FAIR-Checker*

Findability	Accessibility	Reuse (licenses)	Reuse (provenance)
F1B, F2	A1.2	R1.1	R1.2
dct:identifier	odrl:hasPolicy	schema:license	prov:wasGeneratedBy
schema:identifier	dct:rights	dct:license	prov:wasDerivedFrom
dct:title	dct:accessRights	doap:license	prov:wasAttributedTo
dct:description		dbo:license	prov:used
dcat:accessURL		cc:license	prov:wasInformedBy
dcat:downloadURL		xhv:license	prov:wasAssociatedWith
dcat:endpointDescription		sto:license	prov:startedAtTime
dcat:endpointURL		nie:license	prov:endedAtTime
			dct:hasVersion
			dct:isVersionOf
			dct:creator
			dct:contributor
			dct:publisher
			pav:hasVersion
			pav:version
			pav:hasCurrentVersion
			pav:createdBy
			pav:authoredBy
			pav:retrievedFrom
			pav:importedFrom
			pav:createdWith
			pav:retrievedBy
			pav:importedBy
			pav:curatedBy
			pav:createdAt
			pav:previousVersion
			schema:creator
			schema:author
			schema:publisher
			schema:provider
			schema:funder

Specific properties have been proposed to identify resources or concepts such as DC-Terms^15^ or Schema.org^16^*identifier* properties. These properties should be found when assessing the *Findability* principles (F1). In addition, *FAIR-Checker* evaluates if common identification schemes, registered through the *Identifiers.org* [17] resolution service can be found in embedded RDF triples.

One of the reuse criteria (R1.1) lies in making data available with a clearly established access license. A number of ontologies and controlled vocabularies allow to describe licenses in a machine-readable way. For this, we have identified the license properties defined in Schema.org, DC-Terms, DOAP^17^, DBpedia^18^ ontologies.

Another principle of reuse is based on the provision of detailed provenance information (*Reuse* principle R1.2). This information is needed to identify data sources such as authors, funding organizations, but also potential data transformation steps. For this, we selected three commonly used ontologies: PROV^19^ [18], PAV^20^ [19] and DC-Terms. More precisely, they allow to expose time information (e.g. *prov:startedAtTime*, *pav:retrievedOn*), multiple granularity of versioning information (e.g. *pav:hasCurrentVersion*, *pav:previousVersion*), or multiple roles of authorship (e.g. *dct:contributor*, *pav:curatedBy*).

**Fig. 2:**
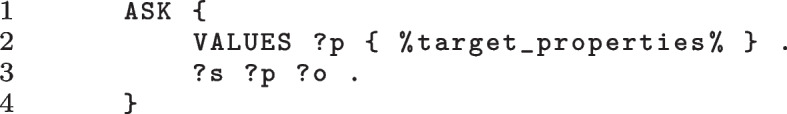
SPARQL ASK query template

For each metrics associated with the FAIR principles described above, we propose to automatically generate, based on a query template, SPARQL ASK queries as shown on Fig. 2.

From a list of target properties, we generate a SPARQL VALUES clause (line 2). When evaluating these queries on the retrieved RDF triples associated to a Web URL, a positive answer is returned when at least one of the predefined target properties can be found.

### Public Knowledge Graphs supporting FAIR assessment

Many public Knowledge Graphs already aggregate, and make accessible, a large number of metadata associated to digital resources such as databases, scientific literature or software. With *FAIR-Checker*, we propose to exploit these semantic data sources during the FAIRification process. From the assessed URL, *FAIR-Checker* generates a SPARQL DESCRIBE query to retrieve the RDF triples already accessible. Wikidata [20] is queried for general knowledge, through the *wikidata:P356* property, used to cross reference digital object identifiers (DOIs). The properties *openaire:resPersistentID* and *datacite:hasIdentifier* are also exploited in SPARQL Describe queries to query respectively the SPARQL endpoints of OpenAIRE and OpenCitation which target scientific literature metadata. As an example, when inspecting Schema.org metadata associated to a scholarly article shared through the Datacite repository, we can add additional metadata to it by retrieving a set of keywords and subjects from the OpenAIRE SPARQL endpoint.

### Handling missing semantic annotations with a generator of profile-based SHACL shapes

Schema.org is a lightweight, general purpose, controlled vocabulary, initially supported by major web search engines, and aimed at semantically annotating web pages. However, since the way major search engines use this metadata is unknown, it is difficult for web data providers to choose which semantic properties to expose, potentially leading to a large diversity of quality in semantic annotations and possibly a lot of missing information.

Community-driven metadata *profiles* have been proposed to tackle this issue. Targeting the Life Sciences community, Bioschemas^21^ [21] is an active community effort supported by the Elixir European Bioinformatics research infrastructure aimed at extending Schema.org and promoting its usage to increase the discoverability of Life Science resources. The Bioschemas community led to more than 37 Schema.org usage recommendations also known as *profiles*. Bioschemas profiles specify which RDF triples should be used to describe specific type of entities. They specify which ontology classes or properties should be used (mostly from Schema.org), enabling the specification of different cardinalities (*one* or *many*), as well as different marginalities (*minimum*, *recommended*, or *optional*) for properties. For instance, the Bioschemas community agreed to state that a web page describing a gene should at least (referring to the *minimum* marginality) provide both *schema:identifier* and *schema:name* properties, but it is recommended (referring to the *recommended* marginality) to also provide a description (*schema:description*) and a reference web page for the gene (*schema:url*).

Up to now, there is no clear consensus on how to represent such profiles with machine-readable formats. With *FAIR-Checker*, we propose to rely on SHACL [22] [23] to automatically represent and evaluate the compatibility of semantic annotations against community-agreed profiles. The marginality of semantic properties is represented with SHACL property shapes. Minimal properties are encoded with a *sh:Violation* severity, whereas recommended properties are encoded with a *sh:Warning* severity.

**Fig. 3:**
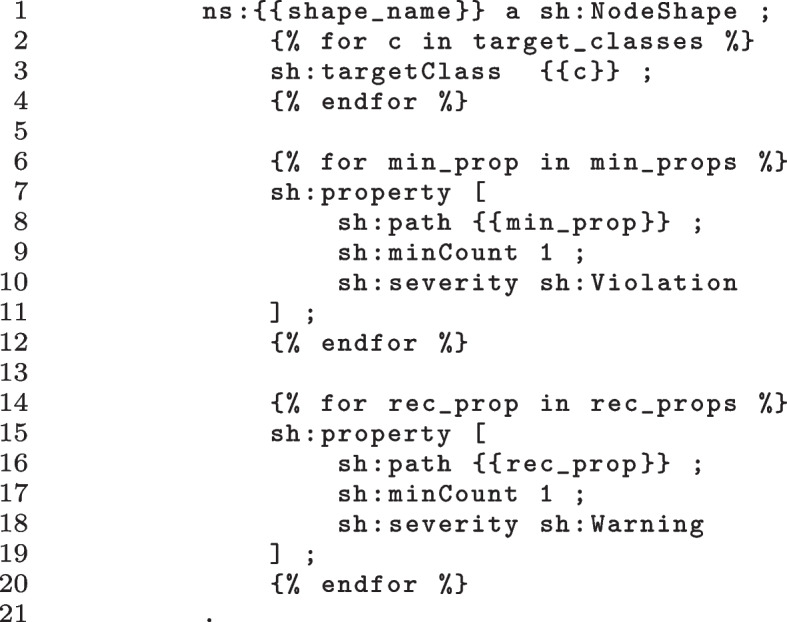
SHACL shape template

Figure 3 shows a generic SHACL shape template. By providing a list of minimal and recommended properties for each Bioschemas profile to a text template engine, *FAIR-Checker* is able to instantiate a profile-specific SHACL shape. Lines 6 to 12 show the iteration over the minimal properties, leading to the generation of multiple property shape patterns specified between lines 7 and 11. The specific property to be evaluated is injected in line 8 through the *min_prop* variable. This pattern is repeated between lines 14 and 20 to address profile-specific recommended properties. The produced shapes are matched against all instances of the *target_class* variables thanks to the iteration specified between lines 2 and 4.

### User recommendations

The reason for a negative result at a given FAIR principle may be difficult to understand for a novice user, and therefore difficult for him to solve. This was a motivation to include a set of easy to understand and accessible recommendations in *FAIR-Checker* , in particular for the “Check” functionality. These recommendations aim to explain how the user can improve its metadata to later validate a failed evaluation. It also provides useful links to training resources such as the FAIR-CookBook^22^. For example, FAIR-CookBook Recipe 1 (Findability section) on unique and persistent identifiers gives the user the necessary background information to assign persistent resource identifiers to its resource and solve a failure on F1B (Persistent IDs) principle. In the same section, FAIR CookBook recipe 8 on Search Engine Optimisation provides examples of structured metadata in JSON-LD, that can help users to encode their metadata in a structured format and solve a failure on F2A (Structured metadata) principle. Also, recipes 3 and 4 in the Interoperability section of the FAIR-CookBook constitute a useful introduction to terminologies and ontologies and a guide for selecting the most appropriate ones. This can be useful to increase compliance with F2B (Shared vocabularies for metadata) and I2B (Machine-readable vocabularies) principles. Finally, concerning the failure due to the lack of license information (R1.1 principle), *FAIR-Checker*  recommendation suggests using one of the following properties: schema:license, dct:license, doap:license, dbpedia-owl:license or cc:license. We are currently collecting user feedback to produce relevant additional recommendations.

## Implementation

*FAIR-Checker* is available at GitHub^23^ under an MIT licence. It is a web application developed in *Python* and based on the *Flask* web framework. *Requests*, *Selenium* and *Extruct* libraries were used to access web pages and extract embedded RDF metadata, while supporting client-side HTML rendering. RDF data, SPARQL queries and SHACL shapes are handled thanks to the *RDFlib* and *pySHACL* libraries.

Object-Oriented techniques were used to foster the extensibility of *FAIR-Checker* to multiple implementations of FAIR metrics. The Factory design pattern allows switching between multiple implementations of the same metrics. An abstract metrics class was designed and thus allowed to implement a generic FAIR metrics evaluation engine.

Code generation techniques, leveraging the *Jinja* template-based text transformation engine, allow adapting multiple and evolving metadata profiles. As shown in Fig. 3 only a single SHACL shape template needs to be maintained for validating multiple Bioschemas profiles. Regarding the selection of Bioschemas profiles for validating specific type of metadata, we first rely on declarations from data providers with the *dct:conformsTo* property, pointing to a Bioschemas profile URL. Since Bioschemas specification repository 24 provides a machine-readable specification of each profiles (JSON-LD), we then generate a SHACL shape for each available profile. When the *dct:conformsTo* is not specified, we propose a candidate profile based on the type (*rdf:type*) of a metadata entity.

We used cache memory techniques to accelerate repetitive processes and alleviate external SPARQL endpoints from re-executing the same query in short periods of time. These caches were implemented through the *flask_caching* and *cachetools* python modules. The former one, of short duration (one minute), is used to store and share RDF metadata for the whole set of metrics, while the second one, updated less frequently (every 2 weeks), is used to store the result of SPARQL queries targeting stable external ontology services (OLS, LOV, and BioPortal) in order to avoid sending multiple identical requests.

**Fig. 4:**
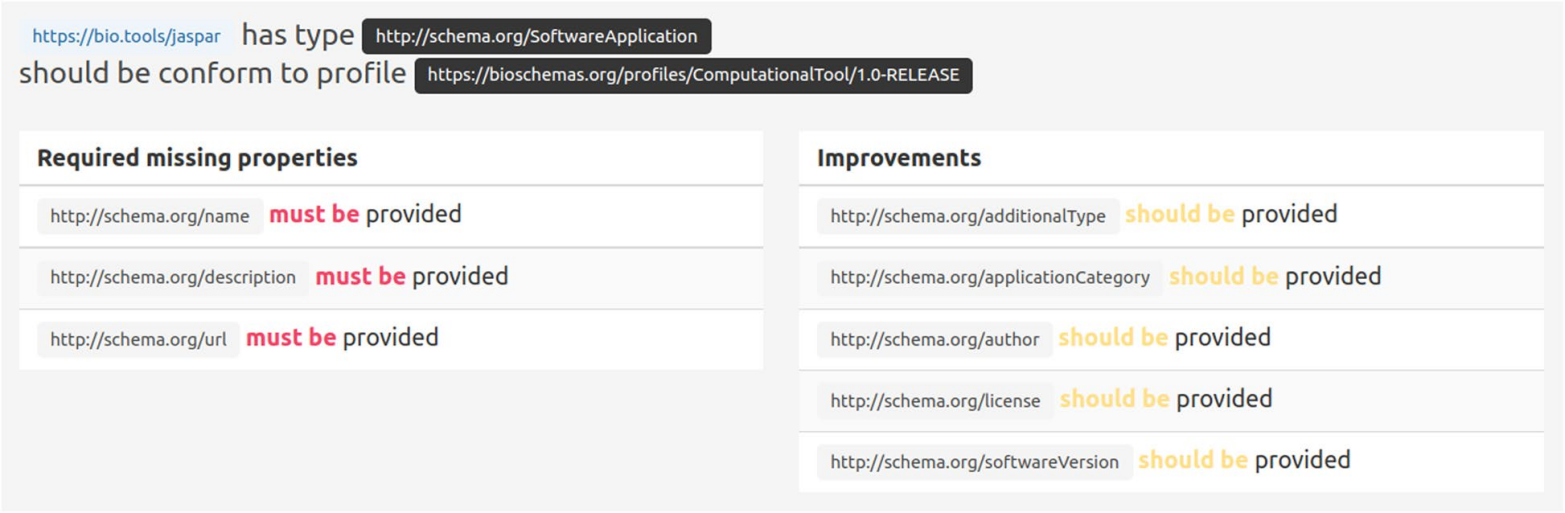
Screenshot of the *FAIR-Checker* user interface reporting metadata completeness results for a Bio.tools software web page against the corresponding Bioschemas *ComputationalTool* profile

Figure 4 shows a sub part of the user interface displaying the result of a SHACL shape evaluation. RDF triples reporting the shape evaluation are queried and transformed into natural language. SHACL *errors* are reported as requirements (“must be”) and *warnings* are reported as improvements (“should be”).

## Results

### Selecting FAIR data repositories

Zenodo, Pangaea and Dryad are repositories aimed at sharing and preserving papers, datasets, software, and research artifacts in general. By identifying resources with DOIs and exposing metadata, they concretely support Open Science. In this experiment, in line with use case UC$_1$, we explore, with *FAIR-Checker* , the potential differences in the way metadata are exposed by several scientific repositories. For each repository, we randomly sampled and harvested 500 web pages describing datasets.

**Fig. 5:**
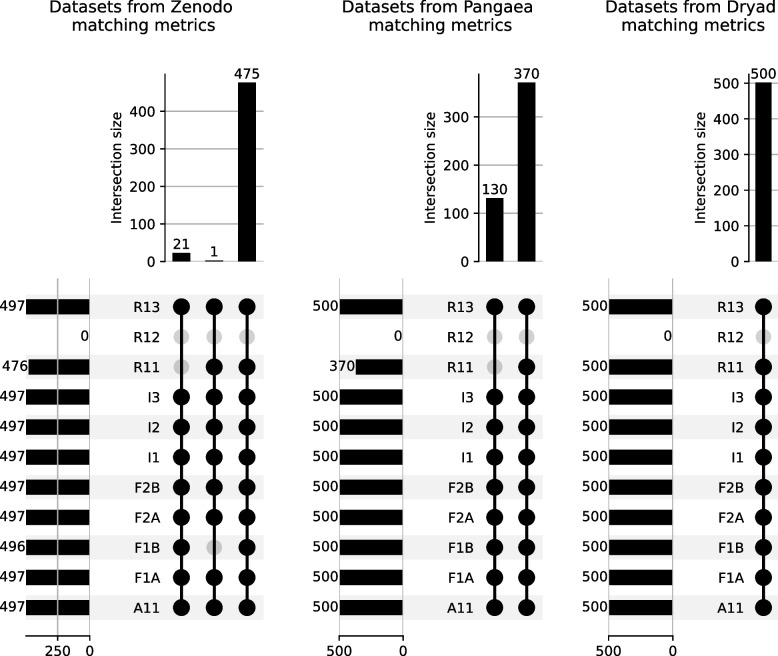
Three UpSet plots comparing the evaluation of the FAIR metrics for dataset web pages served by Zenodo, Pangaea, and Dryad repositories

Figure 5 shows three UpSet^25^ plots aimed at comparing the compliance of the three investigated repositories with the FAIR metrics. Each line represents a metric. A gray point means that the metrics is not validated while a black point means that it is validated. The second line of the left plot shows that none of the datasets in the Zenodo repository validate the R1.2 metric (provenance metadata). The third line shows that, in this repository, 476 of the 497 tested datasets validate the R1.1 metric. Reading these plots vertically, each column counts the number of datasets validating a subset of metrics. The second column of the left plot shows that a single dataset is failing the R1.2 and F1B metrics. The FAIRest resources (475 over 497) are displayed in the rightmost column of this plot where only R1.2 is not validated. The total of 497 datasets for Zenodo instead of 500 for Pangaea and Dryad is explained by the fact that two of them had invalid DOIs wich could not be resolved, while the last one had its DOI property incorrectly formatted. In summary, Fig. 5 shows that none of the three tested repositories expose provenance metadata (R1.2 principle) following the standard vocabularies we identified in Table 2. Although the coverage of FAIR metrics is very similar for these three registries, Dryad seems to be more normative due to the exposition of license metadata for all the 500 randomly sampled resources. This information can be valuable when a data producer wants to select a repository for scientific data sharing (UC$_1$).

### Sharing a software.

**Fig. 6:**
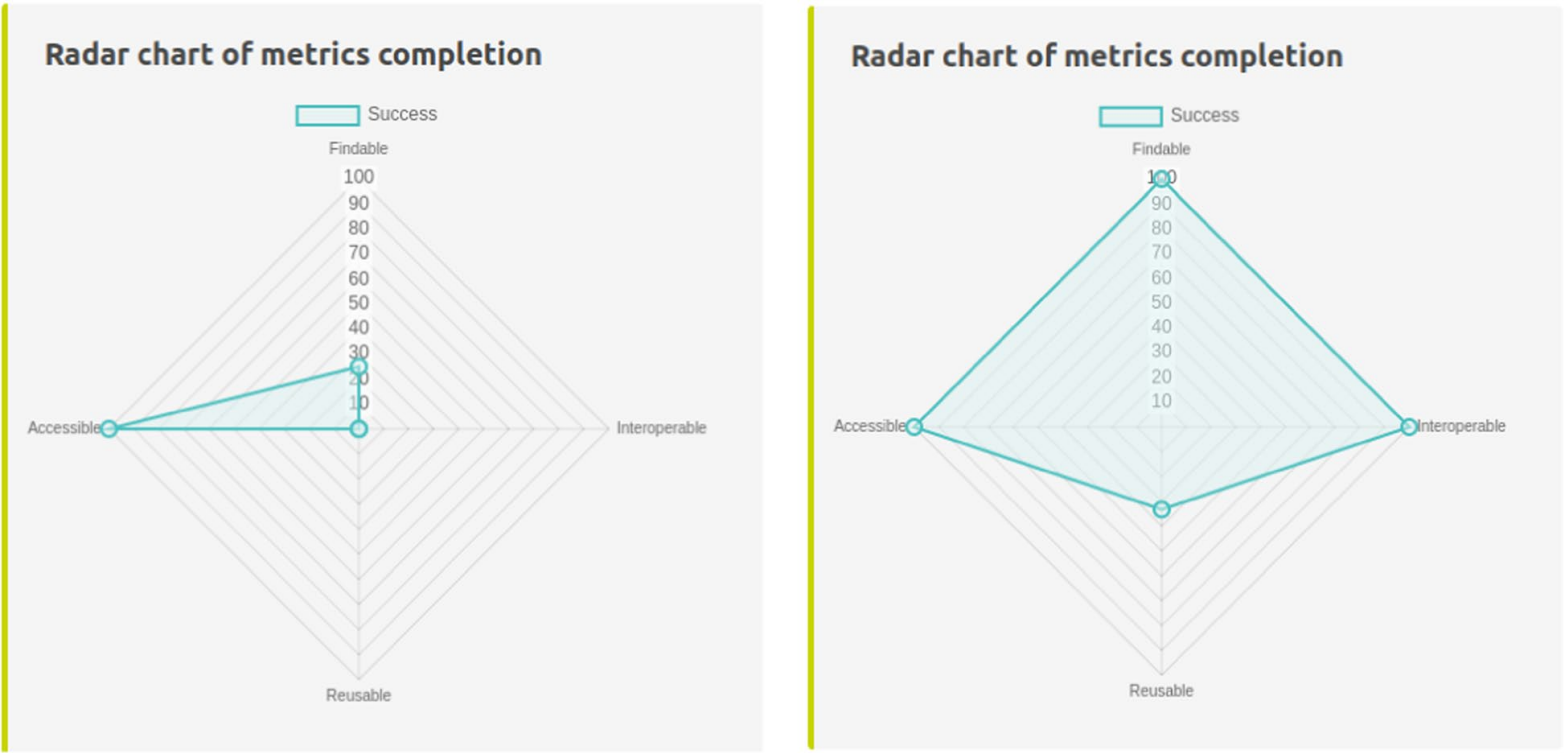
Comparison of the “Check” results on the PhyML landing page before (left) and after (right) the addition of JSON-LD metadata

PhyML [25] is a bioinformatics software aimed at inferring phylogenetic trees. Its website^26^ is a widely used resource for the phylogeneticists community. Its development started when FAIR principles were not as recommended as they are today, leading to a website without embedded metadata. In line with use-case UC$_2$, we used the “Check” function of *FAIR-Checker* to analyze the PhyML early website. As shown in Fig. 6, it complied with only two FAIR metrics (F1.A and A1.1) associated to standard web protocols (URL, HTTP). To improve its FAIRness, we integrated metadata compliant with the Bioschemas *ComputationalTool* profile^27^ for describing a software application. We also used the EDAM bioinformatics ontology [26] to semantically describe i) the disciplines (EDAM Topics) associated to PhyML, ii) the analyses done (EDAM Operations) on input data, and iii) the nature (EDAM Data) of output data. Analysing the new version of the PhyML website with *FAIR-Checker* showed a great improvement: ten metrics were successfully validated. Two metrics (R1.1 and R1.2) are still not validated, making it possible to consider future improvement using the *FAIR-Checker* recommendations described in section “User recommendations”.

### FAIR assessment of a catalog of bioinformatics software.

Bio.Tools is a large community registry cataloging bioinformatics software. In April 2022, the registry had 25, 048 tool descriptions. This registry has been instrumented to expose Schema.org semantic annotations to enhance the tools findability.

In this experiment, we aimed at evaluating the complete collection of 25,048 bioinformatics software descriptions available from Bio.Tools. To speed up the evaluation process, we did not extract the metadata from all individual web pages but analyzed an RDF dataset assembled from all Schema.org annotations provided by the Bio.Tools registry^28^. On a classical laptop workstation, 2.7 GHz quad core Intel Core i7, the whole sequential evaluation of the FAIR metrics - including metrics relying on remote calls to SPARQL endpoints - lasted 42 minutes, which represents a throughput of around 10 evaluations per second.

**Fig. 7:**
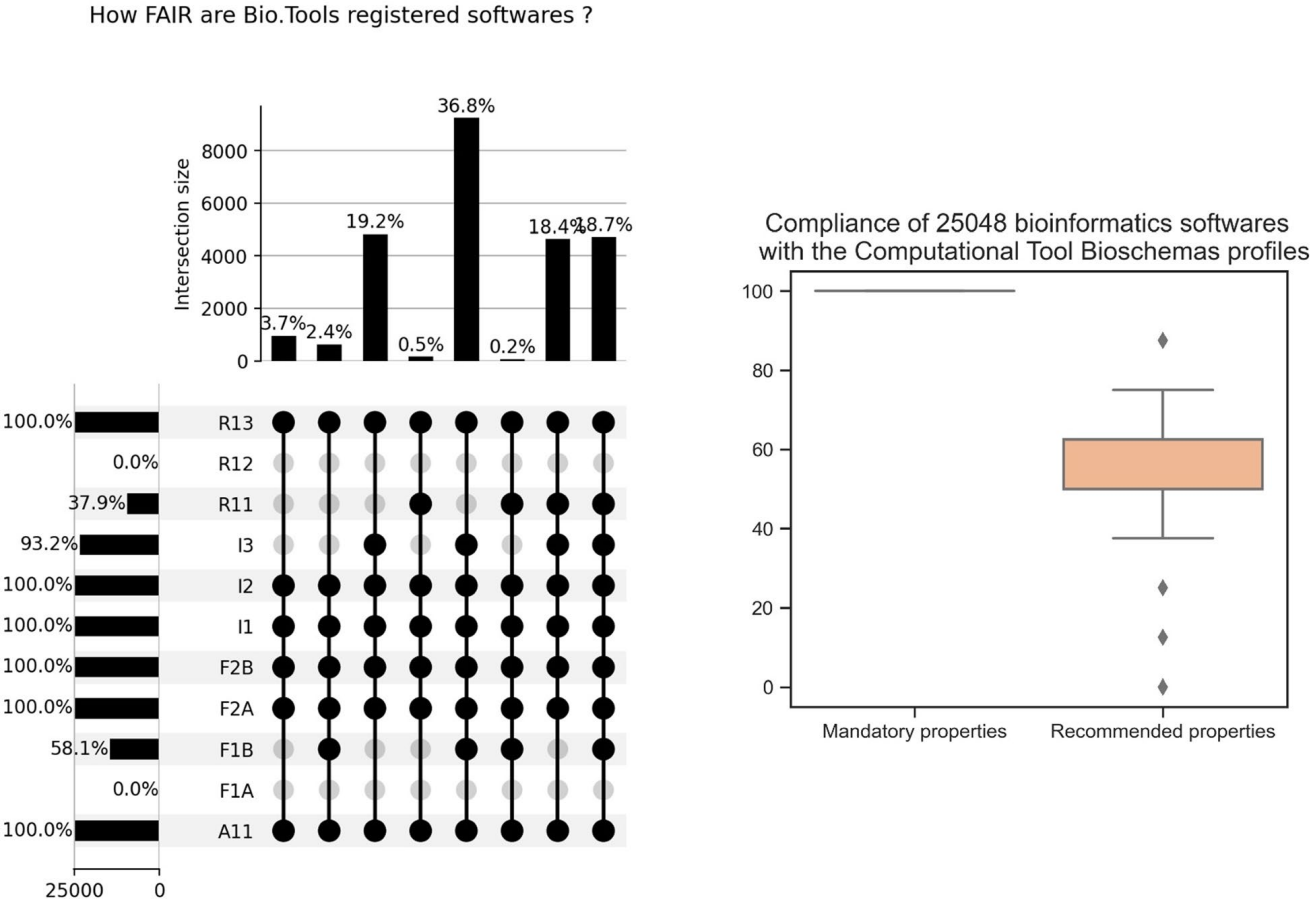
Results of *FAIR-Checker* evaluation on a dump of Schema.org RDF triples associated to 25k+ web pages describing bioinformatics software (left panel) and their compliance with the Bioschemas *ComputationalTool* profile (right panel)

Figure 7 (left panel) summarises the subsets of tools that comply with various combinations of FAIR metrics. The rightmost bar of the histogram (18.7% of the whole collection) represent entries with the largest coverage of FAIR metrics (9/11). We can also note that 36.8% of software descriptions have a good coverage of FAIR metrics (8/11) but do not validate R1.1. From a metadata quality perspective, focusing on the annotation of access licenses would have a significant impact on the FAIRification of these bioinformatics software. The fact that no software description validates the R1.2 metrics highlights the limits of the bio.tools registry. Instrumenting bio.tools to allow the exposition of provenance metadata would have a direct impact on over 25,000 bioinformatics tools.

Finally, in line with use-case UC$_3$, the *FAIR-Checker* “Inspect” function evaluated the Bioschemas *ComputationalTool* profile^29^ on the whole collection of bio.tools entries. On the same laptop configuration as before, the generation and evaluation of SHACL shapes lasted 16 minutes on the whole software collection. The right panel of Fig. 7 reports on the compliance of these entries with respect to both *mandatory* and *recommended* properties. All mandatory properties for computational tools are available for all bio.tools entries. On the other hand, bio.tools provides access to an average of 54% of the recommended properties. These results deserve to be studied in detail by the editors of the bio.tools registry to continue to improve the metadata of the resources it contains.

## Discussion and conclusion

In this paper, we introduce *FAIR-Checker* as a web tool that makes semantic technologies and knowledge graphs accessible to non-expert users, to develop the usage and improve the quality of metadata, thus contributing to the adoption of FAIR principles in practice.

Due to the genericity of FAIR principles and their non-technical specifications, their implementation is highly dependent on human interpretation. We have clarified our technical choices in section “Approach”. Since there is an important overlap between FAIR and Semantic Web principles, we shared some semantic web implementations between multiple FAIR metrics. However, the assessment of a few FAIR principles is kept for future works. The A2 principle^30^, addressing long-term preservation, is clearly out of the scope of Semantic Web technologies. F3^31^ is hard to assess, since there is no consensus on the appropriate vocabulary nor on the associated cardinality constraints aimed at unambiguously identifying data. Regarding I2 (see Table 1), although recent propositions concern the FAIR assessment of ontologies [27–29], to our knowledge, the resulting strategies have not yet been integrated into registries such as BioPortal [30] or FAIRSharing [31].

We are aware that our list of terms to be evaluated by *FAIR-Checker* is limited, albeit founded on common usage in the Semantic Web community as well as more focused efforts such as HCLS [32] or FAIR data points [33]. We propose to capture and discuss specific community needs thanks to GitHub issues that can be directly submitted from *FAIR-Checker*. A more general approach would consist in leveraging crosswalk resources [34] which propose semantic mappings between community specific metadata terms. We envisage as future work to consume these metadata alignments to automate the enrichment of the list of metadata terms considered in *FAIR-Checker*. Specific community efforts such as (e.g. Codemeta crosswalks^32^ is key for an increased adoption During the last 6 monthstools.

During the last 6 months^33^, our national *FAIR-Checker* server^34^ evaluated 28,464 unique resources, and performed 374,595 automated tests. This tool is used as part of the training courses of the French Institute of Bioinformatics, targeting biologists and bioinformaticians. Its national deployment will be accelerated by the high expectations of national programs for the development of Open Sciences. In line with the Bioschemas initiative, which addresses the issues of metadata discoverability, quality and maintenance, FAIR-Checker already targets a large international community of users and developers.

Although the notion of ontologies for the classification of biological objects and their annotation with a controlled terminology is well-known in the Life Science community, the underlying Knowledge Graph standards and technologies, such as RDF, SPARQL or SHACL, are still unfamiliar and difficult to learn. In the context of more open and reproducible sciences, evaluation tools such as *FAIR-Checker* allow building bridges between these technologies and large communities. The genericity of our approach through template SPARQL queries and template SHACL shapes makes it possible to evaluate FAIR principles and more deeply community-specific metadata profiles beyond Life Sciences.

To further promote the adoption of Semantic Web technologies by large communities, progress on identification mechanisms and identity relations must be made. Although this represents a challenging task, an increased use of *sameAs* links in web page metadata could make it easier to exploit public Knowledge Graphs and, as a result, reduce the cost of annotating individual web pages.

The popularity of lightweight ontologies dedicated to resource discovery on the Web (*e.g. Schema.org*) raises concerns about the quality of these semantic annotations, which are widely available and distributed on the Web. The work on metadata profiles and validation (SHEX, SHACL) is particularly interesting in light of the widespread adoption of FAIR principles in Science. In practice, these technologies would allow for the prioritization of the metadata required for resource annotation based on specific community needs. To this aim, we intend to make available the generated SHACL shapes so that they can be inspected by developers, shared on the web or evaluated with other frameworks.

The development of *FAIR-Checker*, as well as initial user feedback, have highlighted important expectations to support the advancement of Open Science. *FAIR-Checker* is still actively developed. A RESTfull API, aimed at serving FAIR assessment for other online tools, has recently been deployed. In future works, we aim at better addressing the diversity of metadata delivery methods, through content negotiation for instance, as well as better and more efficiently leveraging public Knowledge Graphs. We also aim at computing semantic distance with Bioschemas community profiles to suggest relevant profiles, and thus promote their adoption.

## Data Availability

The source code of *FAIR-Checker* is made available from GitHub^35^ under an MIT license. A live FAIR-Checker instance is running online^36^. The Jupyter notebooks and datasets used to produce the experimental result Figures are available at GitHub^37^ and can be re-executed thanks to the MyBinder cloud service.

## Footnotes

1 https://doi.org/10.15497/rda00050

2 https://www.gofair.foundation/interpretation

3 https://fairsharing.github.io/FAIR-Evaluator-FrontEnd

4 https://fairshake.cloud/

5 https://www.fairsfair.eu/f-uji-automated-fair-data-assessment-tool

6 https://www.fairsfair.eu/fair-aware

7 https://ardc.edu.au/resource/fair-data-self-assessment-tool/

8 https://satifyd.dans.knaw.nl

9 https://doi.org/10.5281/zenodo.3922068

10 https://www.go-fair.org/go-fair-initiative/

11 https://fairsharing.github.io/FAIR-Evaluator-FrontEnd

12 Bioportal: https://bioportal.bioontology.org

13 Ontology Lookup Service: https://www.ebi.ac.uk/ols/index

14 Linked Open Vocabularies: https://lov.linkeddata.es/dataset/lov/

15 Dublin Core terms: https://www.dublincore.org/specifications/dublin-core/dcmi-terms/

16 Schema.org: http://schema.org

17 https://github.com/ewilderj/doap

18 https://dbpedia.org/ontology

19 https://www.w3.org/TR/prov-o/

20 https://pav-ontology.github.io/pav/

21 https://bioschemas.org

22 https://faircookbook.elixir-europe.org/content/home.html

23 https://github.com/IFB-ElixirFr/FAIR-checker

24 https://github.com/BioSchemas/specifications

25 An alternative to Venn diagrams for visualizing intersecting sets [24].

26 http://www.atgc-montpellier.fr/phyml

27 https://bioschemas.org/profiles/ComputationalTool/1.0-RELEASE

28 https://github.com/bio-tools/content/tree/master/datasets

29 https://bioschemas.org/profiles/ComputationalTool/1.0-RELEASE

30 *A2: Metadata should be accessible even when the data is no longer available*

31 *F3: Metadata includes the identifier for the data*

32 https://codemeta.github.io/crosswalk/

33 Last statistics computed the 01/06/2023

34 https://fair-checker.france-bioinformatique.fr
